# Protective Effect of Fenofibrate on Oxidative Stress-Induced Apoptosis in Retinal–Choroidal Vascular Endothelial Cells: Implication for Diabetic Retinopathy Treatment

**DOI:** 10.3390/antiox9080712

**Published:** 2020-08-05

**Authors:** Ying-Jung Hsu, Chao-Wen Lin, Sheng-Li Cho, Wei-Shiung Yang, Chung-May Yang, Chang-Hao Yang

**Affiliations:** 1 Graduate Institute of Clinical Medicine, College of Medicine, National Taiwan University, No. 1, Jen Ai Road Section 1, Taipei 100, Taiwan; d98421005@ntu.edu.tw (Y.-J.H.); b91401108@ntu.edu.tw (C.-W.L.); wsyang@ntu.edu.tw (W.-S.Y.); 2 Department of Ophthalmology, National Taiwan University Hospital, No. 7, Zhongshan South Road, Taipei 100, Taiwan; chungmay@ntu.edu.tw; 3 Department of Internal Medicine, National Taiwan University Hospital, No. 7, Zhongshan South Road, Taipei 100, Taiwan; leechoecho@gmail.com

**Keywords:** apoptosis, diabetic retinopathy, fenofibrate, oxidative stress, thioredoxin

## Abstract

Diabetic retinopathy (DR) is an important microvascular complication of diabetes and one of the leading causes of blindness in developed countries. Two large clinical studies showed that fenofibrate, a peroxisome proliferator-activated receptor type α (PPAR-α) agonist, reduces DR progression. We evaluated the protective effects of fenofibrate on retinal/choroidal vascular endothelial cells under oxidative stress and investigated the underlying mechanisms using RF/6A cells as the model system and paraquat (PQ) to induce oxidative stress. Pretreatment with fenofibrate suppressed reactive oxygen species (ROS) production, decreased cellular apoptosis, diminished the changes in the mitochondrial membrane potential, increased the mRNA levels of peroxiredoxin (Prx), thioredoxins (Trxs), B-cell lymphoma 2 (Bcl-2), and Bcl-xl, and reduced the level of B-cell lymphoma 2-associated X protein (Bax) in PQ-stimulated RF/6A cells. Western blot analysis revealed that fenofibrate repressed apoptosis through cytosolic and mitochondrial apoptosis signal-regulated kinase-1 (Ask)-Trx-related signaling pathways, including c-Jun amino-terminal kinase (JNK) phosphorylation, cytochrome c release, caspase 3 activation, and poly (ADP-ribose) polymerase-1 (PARP-1) cleavage. These protective effects of fenofibrate on RF/6A cells may be attributable to its anti-oxidative ability. Our research suggests that fenofibrate could serve as an effective adjunct therapy for ocular oxidative stress-related disorders, such as DR.

## 1. Introduction

Diabetic retinopathy (DR) is a very important microvascular complication of diabetes [1]. It is characterized by a progressive increase in vascular permeability, retinal ischemia and edema, and neovascularization, which results in visual impairment and legal blindness [2]. Retinal vascular endothelial cells play an important role in maintaining the blood-retinal barrier (BRB), which provides a physiological border for retinal homeostasis [3]. Previous studies demonstrated that hyperglycemia induces the activation of oxidative stress and generates reactive oxygen species (ROS) within retinal vascular endothelial cells [4,5]. The accumulation of ROS alters the homeostasis and enhances the migration of retinal vascular endothelial cells, triggers cellular apoptosis, and increases vascular permeability and basement membrane leakage in the retina. These pathological changes may lead to the breakdown of the BRB and DR development [6,7]. Therefore, suppressing oxidative stress could inhibit apoptosis in retinal vascular endothelial cells and reduce the risk of DR progression.

Fenofibrate, a peroxisome proliferator-activated receptor type α (PPAR-α) agonist [8], is used clinically to treat hypertriglyceridemia and hyperlipidemia. However, evidence from two large randomized clinical trials, the Fenofibrate Intervention and Event Lowering in Diabetes (FIELD) and Action to Control Cardiovascular Risk in Diabetes (ACCORD), has shown that fenofibrate significantly prevents DR progression and reduces the use of laser treatment in DR [9,10]. In experimental diabetic models, the expression of PPARα is significantly downregulated in retina [11]. In addition, high glucose medium downregulates PPARα expression in retinal cells [11]. Moreover, over-expression of inflammatory factors, retinal vascular leakage, and more severe DR are found in diabetic PPARα knockout mice [11]. Fenofibrate exerts anti-inflammatory and anti-oxidative effects. Treatment of retinal pigment epithelial cells with fenofibrate reduces high-glucose-induced ROS generation [12]. Fenofibrate downregulated NF-κB, significantly inhibited the expressions of inflammatory mediators and reduced the concentrations of oxidative products in a diabetic rat model [13]. Fenofibrate can significantly reduce lipopolysaccharide (LPS)-induced ROS and increase endothelial nitric oxide (eNOS) levels in human umbilical vein endothelial cells (HUVECs) [14]. Fenofibrate also decreases apoptosis in human retinal endothelial cells and pericytes by activating the AMP-activated protein kinase (AMPK) pathway and downregulating the NF-κB pathway, respectively [15,16]. PPAR-α over-expression or fenofibrate treatment has also been shown to attenuate retinal vascular permeability in diabetic animals [11,17] and may protect against BRB leakage through the down-regulation of basement membrane components [18]. Despite the persuasive results from these clinical and experimental studies, the mechanisms through which fenofibrate protects the eye from DR remain elusive, and further studies are still required to clarify the protective mechanisms of fenofibrate.

Thioredoxins (Trxs) are small proteins that are essential for embryonic development and could protect cells against oxidative stress [19]. There are two main forms of Trx. Trx-1 exists in cytosol, acts as a cofactor of peroxiredoxins (Prx), and plays a direct role in reducing oxidative stress [20,21]. Trx-2 exists in mitochondria and plays an important role in the mitochondrial cellular apoptosis pathway [22,23]. Trxs are essential for life, and Trx gene deficiency is embryonic lethal [24,25]. Trxs are involved in multiple redox-regulated signaling pathways. Trxs bind to apoptosis signal-regulated kinase-1 (Ask-1) in the cytosol and mitochondria, thereby blocking the initiation of the cellular apoptotic process and inhibiting c-Jun amino-terminal kinase (JNK/p38 mitogen-activated protein kinase [MAPK]) [26]. Furthermore, previous studies have shown that Trx is a PPAR-α target gene and that PPAR-α activation induces the translocation of Trx to the nucleus and modulates Trx expression [27]. PPAR-α activator significantly enhances the activation of the Trx promoter and increases Trx-1 expression in human macrophages [28]. However, the role of Trxs against oxidative stress in retinal vascular endothelial cells, as well as the mechanism through which fenofibrate modulates Trxs expression in DR have not been reported.

In this study, we hypothesized that fenofibrate can counteract oxidative stress and attenuate oxidative stress-induced cell apoptosis and death by modulating Trx expression in retinal vascular endothelial cells. Paraquat (PQ) is a common stimulator to induce oxidative stress in in vitro and in vivo studies about retinal degeneration [29,30,31]. Oxidative stress induced by PQ is thought to play an important role in type 2 diabetes through the impairment of insulin action [32,33,34]. Therefore, we used PQ as the inducer of oxidative stress to simulate the condition in DR. We used retinal/choroidal vascular endothelial cell (RF/6A) cells as the cell model system. RF/6A cell line is a monkey choroidal–retinal vascular endothelial cell line and has been widely used to study retinal vascular diseases and DR previously [35,36,37,38,39,40,41,42,43,44]. The study was performed in two parts. First, we investigated the role of oxidative stress in initiating apoptosis and evaluated the protective effects of fenofibrate. Second, we investigated the modulatory effect of fenofibrate on Trx expression and analyzed the related apoptosis and stress signaling pathways.

## 2. Materials and Methods

### 2.1. Cell Culture and Fenofibrate Pretreatment

The RF/6A cell line is a monkey choroidal–retinal vascular endothelial cell line. RF/6A cells were purchased from the American Type Culture Collection (Rockville, MD, USA). RF/6A cells were maintained in Dulbecco’s modified Eagle’s medium (DMEM) with 10% fetal bovine serum, 4.5 mg/mL glucose, 100 units/mL penicillin, and 100 μg/mL streptomycin (all from Thermo Fisher Scientific, Waltham, MA, USA) in a 5% CO_2_ atmosphere at 37 °C. The cells were pretreated with different concentrations of fenofibrate (CAS Number 49562-28-9, Sigma-Aldrich, St. Louis, MO, USA) before exposure to PQ (Sigma-Aldrich, St. Louis, MO, USA).

### 2.2. Cell Viability Assay

The RF/6A cells were seeded at a density of 1 × 10^4^ cells per well onto 96-well plates and incubated at 37 °C. The cells were exposed to 0, 0.2, 0.4, 0.6, 0.8 and 1.0 mM PQ for 24 h. The cells in the fenofibrate treated group were pretreated with 25, 50, 75 or 100 μM fenofibrate for 1 h prior to 24-h exposure of 1.0 mM PQ. 5 mg/mL 3-(4,5-dimethylthiazol-2-yl)-2,5-diphenyltetrazolium bromide (MTT, Chemicon, Millipore, Burlington, MA, USA) was added to each well for 4 h. Then we removed the culture medium supernatant, and formazan was dissolved with dimethyl sulfoxide (DMSO, Sigma-Aldrich, St. Louis, MO, USA) for 30 min at room temperature. The absorbance (570 nm) was measured with a microplate reader (Bio-Rad Laboratories, Hercules, CA, USA).

### 2.3. Analysis of Apoptosis by Flow Cytometry

The RF/6A cells were pretreated with 25, 50, 75 or 100 μM fenofibrate for 1 h prior to 1.0 mM PQ exposure. The proportion of apoptotic RF/6A cells was determined at 24 h by flow cytometry using a staining solution containing 5 μL of annexin-V-FITC and 5 μL of propidium iodide (PI) (Strong Biotech, Taipei, Taiwan) in 250 μL of binding buffer. Cells were washed with PBS and centrifuged at 200 g for 5 min. Then we resuspended the cell pellet in 100 μL of staining solution and incubated for 10 min at 20 °C. Finally, 900 μL of binding buffer was added to the samples, and the samples were analyzed on a FACScan cytometer (BD Bioscience, Franklin Lakes, NJ, USA).

### 2.4. Detection of Intracellular ROS

We measured intracellular ROS levels by 2′,7′-dichlorodihydrofluorescein diacetate (2′,7′-DCFDA, Sigma-Aldrich, St. Louis, MO, USA) oxidation. The RF/6A cells were pretreated with 25, 50, 75 or 100 μM fenofibrate for 1 h prior to 24-h exposure of 1.0 mM PQ. RF/6A cells were then exposed to 10 μM 2′,7′-DCFDA for 10 min. The cells were analyzed by FACScan cytometer (BD Biosciences, Franklin Lakes, NJ, USA) using the FL-1 channel (515–545 nm).

### 2.5. Quantitative Detection of ROS-Induced Cellular Oxidation

The RF/6A cells were pretreated with 25, 50, 75 or 100 μM fenofibrate for 1 h prior to 1.0 mM PQ treatment. After 24-h PQ exposure, DNA oxidation, lipid peroxidation, and protein oxidation levels were determined using an 8-hydroxydeoxyguanosine (8-OHdG) Check Kit (JaICA, Shizuoka, Japan), a thiobarbituric acid reactive substances (TBARS) Assay Kit (Cayman Chemical, Ann Arbor, MN, USA), and a Protein Carbonyl Colorimetric Assay Kit (Cayman Chemical, Ann Arbor, MN, USA), respectively. Cellular DNA was extracted for 8-OHdG detection using a cellular genomic DNA Extraction Kit (T-Pro Biotechenology, New Taipei County, Taiwan). Cellular homogenates were prepared for TBARS or carbonyl colorimetric assays according to the manufacturer’s instructions.

### 2.6. Determination of Mitochondrial Dysfunction

To detect the extent of mitochondrial dysfunction, we measured the mitochondrial membrane potential of cells with JC-1 stain (Cayman Chemical, Ann Arbor, MN, USA). The RF/6A cells were seeded at a density of 1 × 10^4^ cells per well onto 96-well plates and incubated at 37 °C. We added different concentrations of fenofibrate (25, 50, 75, 100 μM) to the cells exposed to 1.0 mM PQ. After a 24-h incubation, 50 μL of JC-1 staining solution buffer was added to 1 mL of culture medium, and the plate was incubated at 37 °C for 15 min. The fluorescence signals for J-aggregates with Texas Red (healthy cells, excitation/emission = 560/595 nm) and JC-1 monomers with FITC (apoptotic or unhealthy cells, excitation/emission = 485/535 nm) were measured with a microplate reader (Bio-Rad Laboratories, Hercules, CA, USA).

### 2.7. Preparation of RNA and cDNA

The RF/6A cells were incubated with 10 μM GW6471 (a PPAR-α antagonist, R&D systems, Minneapolis, MN, USA) for 1 h. After removing GW6471, the cells were then pretreated with 50 or 100 μM fenofibrate for 1 h prior to 1.0 mM PQ treatment. After 24-h PQ exposure, we extracted RNA from RF/6A cells with TRIzol reagent (Thermo Fisher Scientific, Waltham, MA, USA). 1 μg of total RNA was incubated with 300 ng of Oligo dT (Promega, Madison, WI, USA) for 5 min at 65 °C. Samples were then reverse transcribed into cDNA using Moloney murine leukemia virus reverse transcriptase (MMLV-RT; Thermo Fisher Scientific, Waltham, MA, USA) for 1 h at 37 °C. The reaction was terminated by heating the samples for 5 min at 90 °C.

### 2.8. Analysis of mRNA Expression Levels

The resultant cDNA product was subjected to PCR using Prx, Trx-1, Trx-2, B-cell lymphoma 2 (Bcl-2), Bcl-xl, B-cell lymphoma 2-associated X protein (Bax), and β-actin primers. The amplification was performed by thermocycler (MJ Research, Waltham, MA, USA). The 25 μL reaction mixture was composed of 5 μL of cDNA, 200 μM of each deoxynucleotide (DTT), 1 μL of sense and antisense primers, 1.25 U of GoTaq polymerase (Promega, Madison, WI, USA), and 5 μL of 10× Taq polymerase buffer. PCR was performed at an annealing temperature of 56 °C with GoTaq polymerase, cDNA, and the following primers: Prx: 5′-CTTCAGGAAATGCAAAAATTGGGCAT-3′ (forward), 5′-GAGTTTCTTAAATTC TTCTGCTCTA-3′ (reverse); Trx-1: 5′-CCCTTCTTTCATTCCCTCTGTG-3′ (forward), 5′-GAACTCCCCAACCTTTTGACC-3′ (reverse); Trx-2: 5′-CGTACAATGCTGGTGGTCTAAC-3′ (forward), 5′-GTCTTGAAAGTCAGGTCCATCC-3′ (reverse); Bcl-2: 5′-CTGGTGGACAACATCGCTCTG-3′ (forward), 5′-GGTCTGCTGACCTCACTTGTG-3′ (reverse); Bcl-xl: 5′-CCCCAGAAGAAACTGAACCA-3′ (forward), 5′-AGTTTACCCCATCCCGAAAG-3′ (reverse); Bax: 5′-TGGTTGCCCTTTTCTACTTTG-3′ (forward), 5′-GAAGTAGGAAAGGAGGCCATC-3′ (reverse); β-actin: 5′- CTGGAGAAGAGCTATGAGCTG-3′ (forward), 5′- AATCTCCTTCTGCATCCTGTC-3′ (reverse). The DNA fragments were amplified for 25–30 cycles (30 s at 94 °C; 1 min at 50–52 °C; and 1 min at 72 °C), followed by a 7 min extension step at 72 °C. The products were then subjected to electrophoresis on a 1.5% agarose gel and analyzed by gel analyzer system. β-actin was used as the internal control.

### 2.9. Protein Extractions and Western Blot Analysis

The RF/6A cells were incubated with 10 μM GW6471 for 1 h. After removing GW6471, the cells were then pretreated with 50 or 100 μM fenofibrate for 1 h prior to 1.0 mM PQ exposure. After 24-h or 1-h (for phospho-Ask1 and phospho-JNK) PQ exposure, we extracted proteins from RF/6A cells with radioimmunoprecipitation assay (RIPA) lysis buffer, which contained 0.5 M Tris-HCl (pH 7.4), 2.5% deoxycholic acid, 10% NP-40, 1.5 M NaCl, 10 mM EDTA, and 10% protease inhibitors (Complete Mini; Roche Diagnostics, Indianapolis, IN, USA). Mitochondrial proteins and cytosolic proteins were isolated using a mitochondria isolation kit (Thermo Fisher Scientific, Waltham, MA, USA), following the protocol description. For the western blot analysis, the protein samples were separated by a 10% sodium dodecyl sulfate (SDS)-polyacrylamide gel and then transferred to a polyvinylidene difluoride (PVDF) membrane (Immobilon-P; Millipore, Burlington, MA, USA). The primary antibodies used in the experiment were as follows: anti-PPAR-α (at a 1:500 dilution, Santa Cruz Biotechnology, Dallas, TX, USA); anti-Prx-1 (at a 1:1000 dilution, Cell Signaling Technology, Danvers, MA, USA); anti-Trx-1 (at a 1:500 dilution, Cell Signaling Technology, Danvers, MA, USA); anti-Ask-1 (at a 1:1000 dilution, Cell Signaling Technology, Danvers, MA, USA); anti-phospho-Ask1 (at a 1:2000 dilution, Bioss, Woburn, MA, USA); anti-JNK (at a 1:3000 dilution, Cell Signaling Technology, Danvers, MA, USA); anti-phospho-JNK (at a 1:3000 dilution, Cell Signaling Technology, Danvers, MA, USA); anti-Bcl-2 (at a 1:1000 dilution, Cell Signaling Technology, Danvers, MA, USA); anti-Bcl-xl (at a 1:500 dilution, Cell Signaling Technology, Danvers, MA, USA); anti-Bax (at a 1:5000 dilution, Cell Signaling Technology, Danvers, MA, USA); anti-cytochrome c (at a 1:1000 dilution, Abcam, Hong Kong, China); anti-VDAC1 (at a 1:5000 dilution, Abcam, Hong Kong, China); anti-Trx-2 (at a 1:2000 dilution, R&D System, Minneapolis, MN, USA,); anti-apoptotic protease activating factor-1 (Apaf-1) (at a 1:1000 dilution, Cell Signaling Technology, Danvers, MA, USA); anti-caspase-9 (at a 1:1000 dilution, Cell Signaling Technology, Danvers, MA, USA); anti-caspase-7 (at a 1:1000 dilution, Cell Signaling Technology, Danvers, MA, USA); anti- poly (ADP-ribose) polymerase-1 (PARP-1) (at a 1:1000 dilution, Abcam, Hong Kong, China); and anti-β-actin (at a 1:5000 dilution, Bioss, Woburn, MA, USA). Immunodetections were performed using enhanced chemiluminescence (Pierce Biotechnology, Waltham, MA, USA). Protein levels were determined using densitometry analysis of the protein bands. Protein levels were normalized to β-actin.

### 2.10. Caspase-3 Activity Assay

The RF/6A cells were incubated with 10 μM GW6471 for 1 h. After removing GW6471, the cells were then pretreated with 50 or 100 μM fenofibrate for 1 h prior to 1.0 mM PQ exposure. After 24 h PQ exposure, the caspase-3 activity of RF/6A cells was analyzed by the Caspase-3/CPP32 Colorimetric Assay Kit (BioVision, Milpitas, CA, USA). Assay procedures were performed following the manufacturer’s instructions.

### 2.11. Statistical Analyses

The results are expressed as mean ± standard deviation. We used Mann–Whitney U-test to compare the data between two groups. We used Kruskal–Wallis test with post hoc Dunn’s test to compare the data among multiple different groups. P values of less than 0.05 were considered statistically significant. Statistical analysis was performed using SPSS (version 17.0, SPSS, Chicago, IL, USA).

## 3. Results

### 3.1. Fenofibrate Treatment Decreased PQ-Induced RF/6A Cell Death

**Figure 1:**
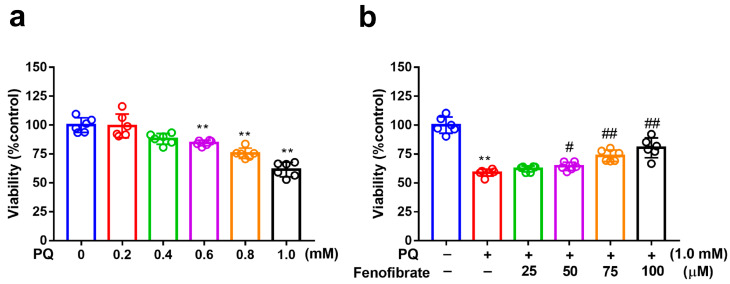
Effects of fenofibrate on cell viability in paraquat (PQ)-stimulated RF/6A cells assessed using MTT assay. (**a**) Cell viability after exposure to different concentrations of PQ for 24 h. (** *p* < 0.01 among the control group and 0.6, 0.8, and 1.0 mM PQ-stimulated groups using Kruskal–Wallis test with post hoc Dunn’s test; *n* = 6 in each group) (**b**) Cell viability in PQ-stimulated RF/6A cells with fenofibrate pre-treatment. RF/6A cells were pretreated with different concentration of fenofibrate for 1 h, then exposed to 1.0 mM PQ for 24 h. (** *p* < 0.01 between the control group and 1.0 mM PQ-stimulated group using Mann–Whitney U-test; # *p* < 0.05, ## *p* < 0.01 compared to only 1.0 mM PQ-stimulated group using Kruskal–Wallis test with post hoc Dunn’s test; *n* = 6 in each group).

MTT assay was used to evaluate cell viability. After exposure to several concentrations of PQ for 24 h, the viability of RF/6A cells reduced to 88%, 77%, and 60% at PQ concentrations of 0.6 mM, 0.8 mM, and 1.0 mM, respectively (Figure 1a). Viability decreased substantially after exposure to 1.0 mM PQ. Therefore, we chose 1.0 mM as the concentration of PQ in the following experiments. When the cells were pretreated with fenofibrate and then exposed to 1.0 mM PQ, the survival rate increased in a dose-dependent manner (from 65% in only PQ-stimulated group to 83% at 100 µM fenofibrate) (Figure 1b).

### 3.2. Fenofibrate Treatment Suppressed PQ-Induced Apoptosis in RF/6A Cells

**Figure 2:**
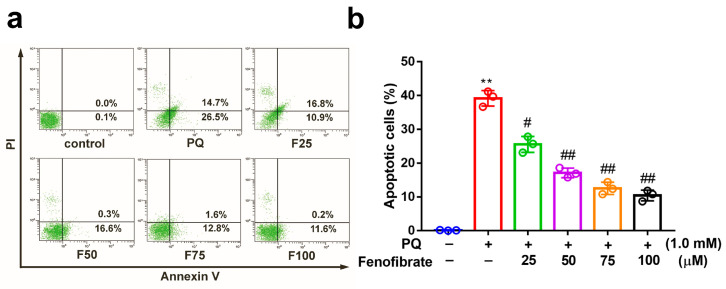
Effects of fenofibrate on apoptosis in paraquat (PQ)-stimulated RF/6A cells assessed by flow cytometry. (**a**) RF/6A cells were pretreated with different concentrations of fenofibrate for 1 h and then exposed to 1.0 mM PQ for 24 h. The *x*-axis and *y*-axis represent annexin V-FITC and propidium iodide (PI) staining, respectively. PQ: 1.0 mM PQ; F25: 1.0 mM PQ with 25 µM fenofibrate; F50: 1.0 mM PQ with 50 µM fenofibrate; F75: 1.0 mM PQ with 75 µM fenofibrate; F100: 1.0 mM PQ with 100 µM fenofibrate. (**b**) Percentage of apoptotic cells treated with different concentrations of fenofibrate. (** *p* < 0.01 between the control group and 1.0 mM PQ-stimulated group using Mann–Whitney U-test; # *p* < 0.05, ## *p* < 0.01 compared to only 1.0 mM PQ-stimulated group using Kruskal–Wallis test with post hoc Dunn’s test; *n* = 3 in each group).

We investigated the effects of fenofibrate on PQ-stimulated cell apoptosis by flow cytometry. After exposure to 1.0 mM PQ, the level of cell apoptosis was significantly increased compared to that in the control group. Prior treatment with fenofibrate before PQ stimulation protected RF/6A cells and dose-dependently decreased the levels of cell apoptosis (Figure 2).

### 3.3. Fenofibrate Treatment Suppressed PQ-Induced ROS, 8-OHdG, Malondialdehyde, and Protein Carbonyl Content Production in RF/6A Cells

**Figure 3:**
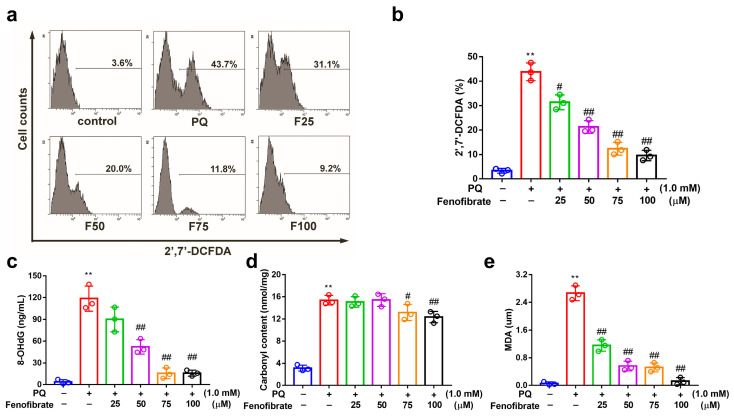
Effects of fenofibrate on reactive oxygen species (ROS) production and oxidative stress indicators in paraquat (PQ)-stimulated RF/6A cells assessed by flow cytometry. RF/6A cells were pretreated with different concentration of fenofibrate for 1 h, then exposed to 1 mM PQ for 24 h. (**a**) PQ-induced ROS production under fenofibrate treatment. The *x*-axis represents 2′,7′-dichlorodihydrofluorescein diacetate (2′,7′-DCFDA) staining, and the *Y*-axis represents cell numbers. PQ: 1 mM PQ; F25: 1 mM PQ with 25 µM fenofibrate; F50: 1 mM PQ with 50 µM fenofibrate; F75: 1 mM PQ with 75 µM fenofibrate; F100: 1 mM PQ with 100 µM fenofibrate. Dose-dependent effect of fenofibrate treatment on (**b**) ROS production; (**c**) the expression of 8-hydroxydeoxyguanosine (8-OHdG), a DNA oxidation indicator; (**d**) the expression of malondialdehyde (MDA), a lipid peroxidation indicator; (**e**) the expression of protein carbonyl content, a protein oxidation indicator. (** *p* < 0.01 between the control group and 1 mM PQ-stimulated group using Mann–Whitney U-test; # *p* < 0.05, ## *p* < 0.01 compared to only 1 mM PQ-stimulated group using Kruskal–Wallis test with post hoc Dunn’s test; *n* = 3 in each group).

PQ stimulation can induce oxidative stress by overproducing ROS in RF/6A cells. PQ stimulation led to an increased ROS production, which was reduced by pretreatment with fenofibrate (Figure 3a,b). To further investigate the effects of fenofibrate on oxidative stress, the levels of 8-OHdG (oxidative DNA adduct), malondialdehyde (MDA, lipid peroxidation product), and protein carbonyl content (protein oxidative marker) were evaluated. The levels of 8-OHdG, MDA and protein carbonyl content were significantly increased after exposure to PQ. The levels of 8-OHdG and MDA decreased with fenofibrate pretreatment in a dose-dependent manner (Figure 3c,d). The levels of protein carbonyl content were reduced with higher concentration of fenofibrate (75 and 100 µM) (Figure 3e).

### 3.4. Fenofibrate Treatment Diminished Mitochondrial Damage in PQ-Induced RF/6A Cell

**Figure 4:**
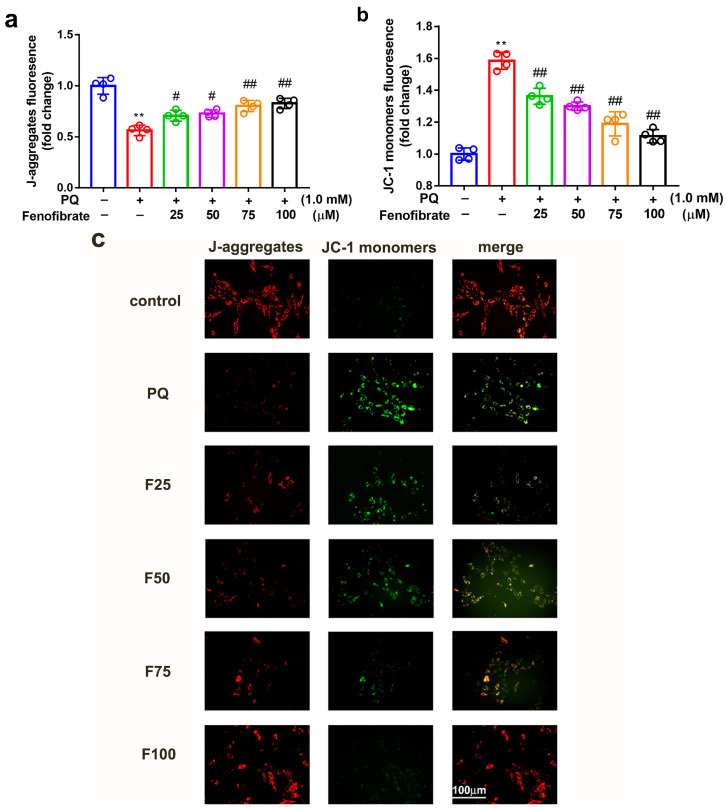
Effects of fenofibrate on mitochondrial damage in RF/6A cells assessed by JC-1 staining. RF/6A cells were pretreated with different concentrations of fenofibrate for 1 h, then exposed to 1 mM paraquat (PQ) for 24 h. Dose-dependent effect of fenofibrate treatment on (**a**) the expression of J-aggregates in PQ-stimulated RF/6A cells, and (**b**) JC-1 monomers in PQ-stimulated RF/6A cells. (** *p* < 0.01 between the control group and 1 mM PQ-stimulated group using Mann–Whitney U-test; # *p* < 0.05, ## *p* < 0.01 compared to only 1 mM PQ-stimulated group using Kruskal–Wallis test with post hoc Dunn’s test; *n* = 4 in each group) PQ: 1 mM PQ; F25: 1 mM PQ with 25 µM fenofibrate; F50: 1 mM PQ with 50 µM fenofibrate; F75: 1 mM PQ with 75 µM fenofibrate; F100: 1 mM PQ with 100 µM fenofibrate. (**c**) Fluorescence microscopy images showing the expression of JC-1 monomers (FITC) and J-aggregates (Texas Red).

To determine whether fenofibrate can protect mitochondrial function, the extent of mitochondrial damage was analyzed using a JC-1 assay. JC-1 spontaneously formed J-aggregates in healthy cells. Our results showed that PQ stimulation significantly decreased the ratio of J-aggregates compared to that in control group. Fenofibrate treatment dose-dependently increased the expression of J-aggregates in RF/6A cells (Figure 4a). JC-1 remained in the monomeric form in apoptotic or unhealthy cells. After PQ exposure, the expression of JC-1 monomers had a 1.58-fold increase compared to that in control group. Fenofibrate treatment decreased the expression of JC-1 monomers in a dose-dependent manner (Figure 4b). The fluorescence signal revealed a high level of JC-1 monomers (FITC) in PQ-stimulated cells; conversely, a high level of J-aggregates (Texas Red) was detected in the control group. Fenofibrate pretreatment decreased the level of JC-1 monomers and increased the level of J-aggregates in a dose-dependent manner (Figure 4c).

### 3.5. Effects of Fenofibrate on PQ-Induced Oxidative Stress-Related mRNA Levels in RF/6A Cells

**Figure 5:**
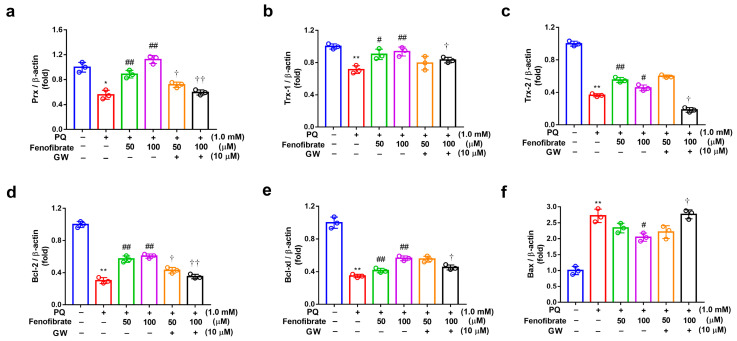
mRNA expression of peroxiredoxin (Prx), thioredoxin-1 (Trx-1), Trx-2, B-cell lymphoma 2 (Bcl-2), Bcl-xl, and B-cell lymphoma 2-associated X protein (Bax) in RF/6A cells detected using semi-quantitative PCR. RF/6A cells were pretreated with a high or low dose of fenofibrate or 1 h, then stimulated with 1 mM paraquat (PQ) for 24 h. In GW6471 (GW) treated groups, the cells were incubated with 10 μM GW6471 for 1 h before fenofibrate treatment. (**a**) Relative expression of Prx. (**b**) Relative expression of Trx-1. (**c**) Relative expression of Trx-2. (**d**) Relative expression of Bcl-2. (**e**) Relative expression of Bcl-xl. (**f**) Relative expression of Bax. (* *p* < 0.05, ** *p* < 0.01 between the control group and 1 mM PQ-stimulated group using Mann–Whitney U-test; # *p* < 0.05, ## *p* < 0.01 compared to only 1 mM PQ-stimulated group using Kruskal–Wallis test with post hoc Dunn’s test; † *p* < 0.05, †† *p* < 0.01 between GW6471 treated group and fenofibrate treated group (the same concentration of fenofibrate) using Mann–Whitney U-test; *n* = 3 in each group; β-actin was used as an internal control.).

The mRNA levels of Prx, Trx-1, Trx-2, Bcl-2, Bcl-xl, and Bax were determined using semi-quantitative PCR analysis (Figure 5). Compared to those of the control group, the expression levels of Prx, Trx-1, Trx-2, Bcl-2, and Bcl-xl mRNA were significantly lower in the PQ-stimulated group. Fenofibrate treatment significantly enhanced the expression of Prx, Trx-1, Bcl-2, and Bcl-xl mRNA levels in a dose-dependent manner (Figure 5a–e). However, the increase of Trx-1 expression was not concentration-dependent (Figure 5c). The mRNA level of Bax was significantly higher in the PQ-stimulated group than that in control group. Only high-dose fenofibrate reduced Bax mRNA level (Figure 5f). To further confirm the effects of fenofibrate, a PPAR-α antagonist, GW6471, was added to the medium before fenofibrate treatment. The results revealed that 10 µM GW6471 could attenuate the effect of fenofibrate on Prx, Trx-1, Trx-2, Bcl-2, Bcl-xl, and Bax mRNA expression (Figure 5a–f).

### 3.6. Effects of Fenofibrate on PQ-Induced Apoptosis and Stress-Signaling Pathway-Related Proteins in RF/6A Cells

**Figure 6:**
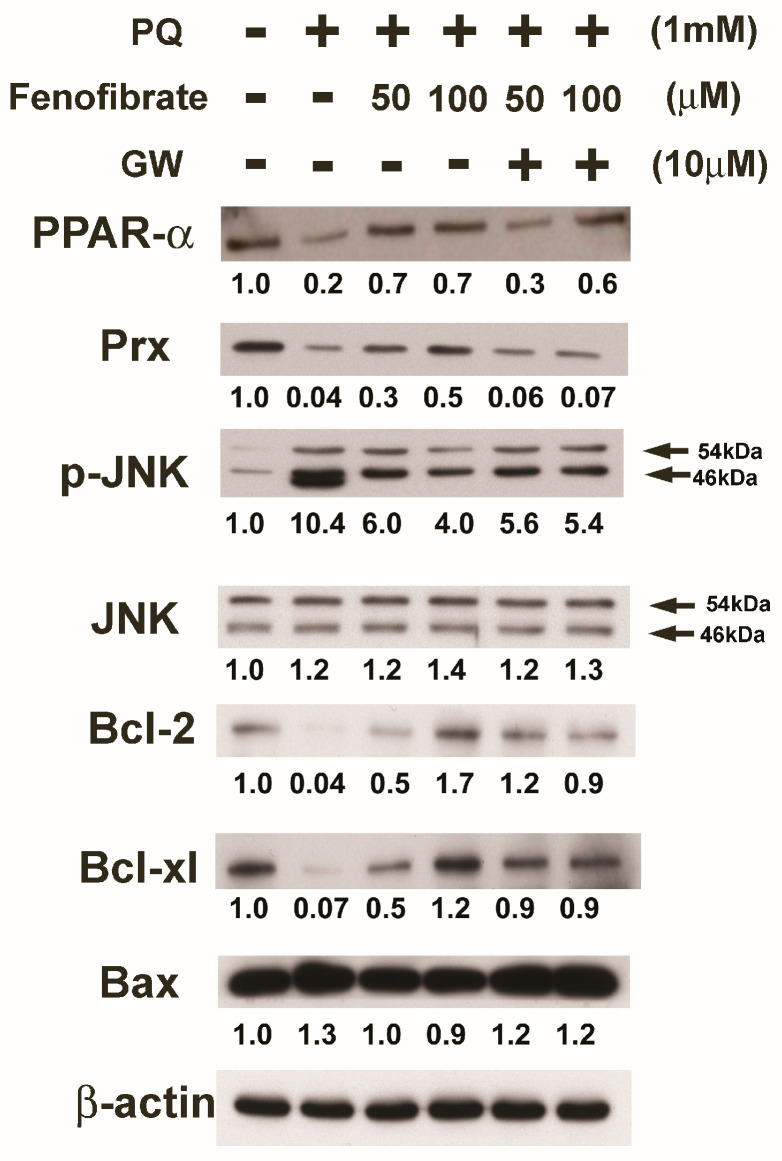
Effects of fenofibrate on the expression of paraquat (PQ)-induced apoptosis and stress-signaling pathway-related proteins assessed by western blot analysis. RF/6A cells were pretreated with a high or low dose of fenofibrate for 1 h, then exposed to 1 mM PQ for 1 h (for phospho-c-Jun amino-terminal kinase (p-JNK)) or 24 h. In GW6471 (GW) treated groups, the cells were incubated with 10 μM GW6471 for 1 h before fenofibrate treatment. The expression levels of peroxisome proliferator-activated receptor type α (PPAR-α), peroxiredoxin (Prx), p-JNK, JNK, B-cell lymphoma 2 (Bcl-2), Bcl-xl, and B-cell lymphoma 2-associated X protein (Bax) are shown and the fold changes compared to those in control group are presented under the protein bands. β-actin was used as an internal control.

We evaluated the effects of fenofibrate on PQ-induced apoptosis and stress-signaling pathway-related proteins in RF/6A cells. PQ stimulation decreased the expression of PPAR-α, Prx, Bcl-2, and Bcl-xl compared to that of the control group. The expression of PPAR-α, Prx, Bcl-2, and Bcl-xl increased with fenofibrate pretreatment. The expression of p-JNK and Bax increased after PQ exposure and was suppressed by fenofibrate pretreatment. The effects of fenofibrate were partially counteracted by 10 μM of GW6471 (Figure 6).

**Figure 7:**
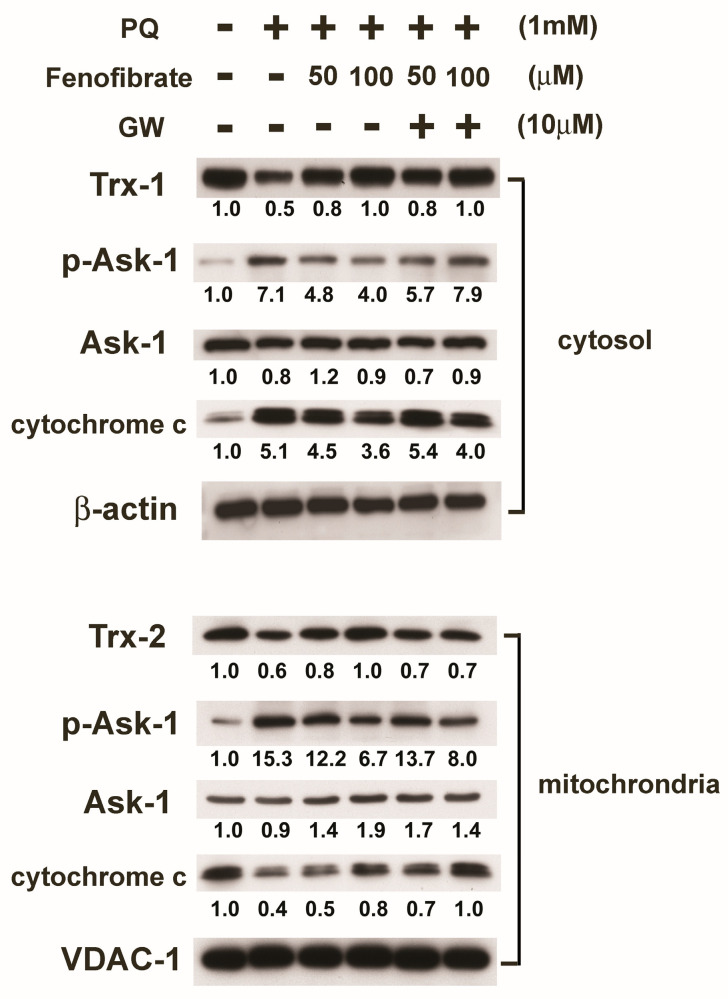
Effects of fenofibrate on the expression of paraquat (PQ)-induced thioredoxins (Trxs), apoptosis signal-regulated kinase-1 (Ask-1), and cytochrome c assessed by western blot analysis. RF/6A cells were pretreated with a high or low dose of fenofibrate for 1 h, then exposed to 1 mM PQ for 1 h (for phospho-Ask-1 (p-Ask-1)) or 24 h. In GW6471 (GW) treated groups, the cells were incubated with 10 μM GW6471 for 1 h before fenofibrate treatment. Mitochondrial proteins and cytosolic proteins were isolated and analyzed separately. The expression levels of mitochondrial Trx-2, Ask-1, p-Ask-1, and cytochrome c and cytosolic Trx-1, Ask-1, p-Ask-1, and cytochrome c are shown, and the fold changes compared to those in control group are presented under the protein bands. In cytosol, β-actin was used as an internal control. In mitochondria, VDAC-1 was used as an internal control.

We then assessed protein expression in mitochondria and cytosol. In mitochondria, PQ stimulation enhanced p-Ask-1 expression but reduced cytochrome c and Trx-2 expression compared to that of the control group. Fenofibrate treatment enhanced cytochrome c and Trx-2 expression and suppressed p-Ask-1 expression. Stimulation of PQ facilitated cytochrome c release from the mitochondria into cytosol, and fenofibrate treatment inhibited the release of cytochrome c. In addition, PQ stimulation enhanced p-Ask-1 expression but reduced Trx-1 expression in cytosol. Fenofibrate treatment enhanced Trx-1 expression and suppressed p-Ask-1 expression in cytosol. The effects of fenofibrate were also partially counteracted by 10 μM of GW6471 (Figure 7).

**Figure 8:**
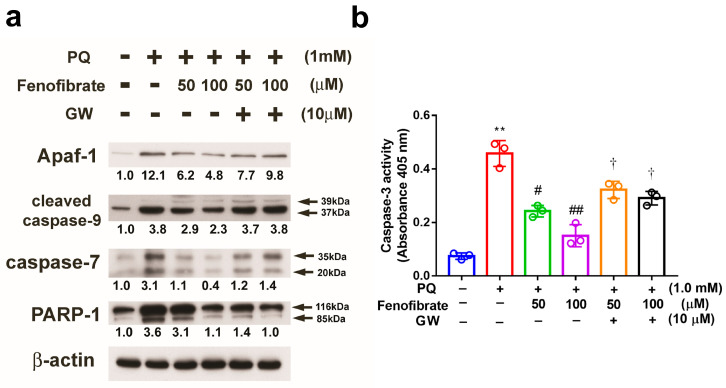
Effects of fenofibrate on the expression of paraquat (PQ)-induced apoptosis-related proteins assessed by western blot analysis. RF/6A cells were pretreated with a high or low dose of fenofibrate for 1 h, then exposed to 1 mM PQ for 24 h. In GW6471 (GW) treated groups, the cells were incubated with 10 μM GW6471 for 1 h before fenofibrate treatment. (**a**) The expression levels of anti-apoptotic protease activating factor-1 (Apaf-1), cleaved caspase-9, caspase-7, and poly (ADP-ribose) polymerase-1 (PARP-1) are shown. The fold changes compared to those in control group are presented under the protein bands. β-actin was used as an internal control. (**b**) Caspase-3 activity. (** *p* < 0.01 between the control group and 1 mM PQ-stimulated group using Mann–Whitney U-test; # *p* < 0.05, ## *p* < 0.01 compared to only 1 mM PQ-stimulated group using Kruskal–Wallis test with post hoc Dunn’s test; † *p* < 0.05 between GW6471 treated group and fenofibrate treated group (the same concentration of fenofibrate) using Mann–Whitney U-test; *n* = 3 in each group.).

PQ stimulation enhanced the expression of Apaf-1, cleaved caspase-9, and caspase-7 compared to that in control group, and the expression levels of these proteins were suppressed by fenofibrate treatment. The effects of fenofibrate were partially counteracted by 10 μM of GW6471. PARP-1 was cleaved in PQ-stimulated cells, and the level of cleavage form of PARP-1 was diminished by fenofibrate treatment (Figure 8a). We also assessed the activity of caspase-3 and the results demonstrated that PQ stimulation significantly increased caspase-3 activity. The activity of caspase-3 was inhibited by fenofibrate treatment in a dose-dependent manner. The effects of fenofibrate were also partially counteracted by the addition of 10 µM GW6471 (Figure 8b).

## 4. Discussion

In the present study, we demonstrated the protective effects of fenofibrate on RF/6A cells under oxidative stress. Fenofibrate inhibited ROS accumulation, mitochondrial dysfunction, and modulated the apoptosis and stress signaling pathway in oxidative stress-induced RF/6A cells.

Increasing evidence supports the idea that oxidative stress plays an important role in the pathogenesis of DR. PPAR-α is a regulator of inflammation and oxidative stress that induces the activation of antioxidant enzymes [45,46,47]. Evidence suggests that fenofibrate may modulate anti-oxidant pathways. For example, fenofibrate inhibits the production of ROS in streptozotocin-induced diabetic rats and reduces nephropathy development [48]. In the present study, the mRNA expression of anti-oxidant enzymes Prx, Trx1, and Trx-2 decreased in PQ-stimulated RF/6A cells, whereas the mRNA levels of these enzymes increased after fenofibrate treatment. This finding indicated that fenofibrate may induce the expression of anti-oxidant proteins and protect cells from oxidative stress. Endothelial cell apoptosis has been linked to oxidative damage through the production of 8-OHdG, nitrotyrosine, and MDA [49,50]. In the present study, the results showed that fenofibrate suppressed MDA production and protected vascular endothelial cells from lipid peroxidation. We also observed that fenofibrate suppressed 8-OHdG adduct formation but only inhibited protein oxidation at higher concentrations. Previous studies have also revealed that fenofibrate could suppress MDA production in rat models for low-density lipoprotein-induced endothelial dysfunction and Parkinson’s disease [51,52]. Taken together, the results from the present study suggested that fenofibrate could induce the expression of anti-oxidant enzymes, reduce the production of ROS and decrease the generation of oxidant products, thus protecting endothelial cells from oxidative stress-induced damage.

Mitochondria are a major source of oxidative stress in DR because oxidative stress in the inner membrane leads to imbalance in the electron transport chain and generates superoxide and hydrogen peroxide, thereby damaging the membrane proteins. Furthermore, mitochondrial dysfunction activates the apoptosis-related signaling pathway [53]. Fenofibrate has been reported to decrease apoptosis in high-glucose-stimulated microvascular endothelial cells [54] and decrease the apoptotic rate of the ganglion cells in the mouse model for type 2 diabetes [55]. In the present study, we observed that fenofibrate reduced the apoptotic rate and could preserve mitochondrial function in PQ-stimulated RF/6A cells. Our findings suggested that fenofibrate could inhibit cell death and DR progression by preventing mitochondrial dysfunction.

Trxs belong to a group of small redox proteins that can be found in most cells. The anti-oxidative activity of Trxs is indispensable for cells [56]. Trxs exert most anti-oxidant properties in cells through thioredoxin peroxidase [18]. Niso-Santano et al. observed that PQ induces the phosphorylation of Ask-1 and suppress Trx expression in SH-SY5Y cells (human neuroblastoma cells) [57]. Trx-1 levels are also reduced in mycophenolic acid-induced apoptosis in pancreatic β-cells [58]. Fiuza et al. demonstrated that the protective effects of diphenyl diselenide on endothelial cells against oxidative stress are through the expression of different isoforms of Prx [59]. In our study, we found that the mRNA and protein expression of Prx, Trx-1, and Trx-2 decreased, and phosphorylated Ask-1 increased in PQ-stimulated RF/6A cells. In addition, thioredoxin-interacting protein (TRXIP) was reported to be significantly up-regulated in DR. TRXIP may interact with Trx, block its anti-oxidant activity, and then cause mitochondrial dysfunction and inflammation in DR [60,61]. The expression of Trx increased after fenofibrate treatment in our experiments. Our results were consistent with that of the study conducted by Billiet et al., in which PPAR-α activation induced Trx-1 expression [28]. The addition of PPAR-α antagonist could attenuate but not completely abolish the effects of fenofibrate, indicating that the effects of fenofibrate were not all PPAR-α dependent. In summary, our study suggested that the anti-oxidative activity and anti-apoptotic effects of fenofibrate could be attributed to the increase of Trx expression and the inhibition of Ask-1 phosphorylation.

We then investigated the effects of fenofibrate on the regulation of Trx-related signaling pathways. Trx binds to Ask-1 in the mitochondria and cytosol, thereby blocking the initiation of the cellular apoptotic process and inhibiting the activation of JNK/p38 MAP kinase [26]. In the cytosol, Ask-1 is required for the activation of JNK/p38 MAP kinases. Bcl-2 and Bcl-xl are known to regulate mitochondrial dynamics and play essential roles in anti-apoptosis; however, Bax promotes apoptosis [62,63]. JNK/p38 MAP kinase also regulates mitochondrial-mediated apoptosis [64] and facilitates the release of mitochondrial cytochrome c to the cytosol. Our study revealed that p-JNK and Bax expression were elevated in PQ-stimulated RF/6A cells and fenofibrate treatment suppressed their expression. Conversely, the expression of Bcl-2 and Bcl-xl increased after fenofibrate treatment. In mitochondria, Trx-2 inhibits Ask-1-mediated apoptosis, which in turn causes the inhibition of cytochrome c release to the cytosol [65]. Our findings showed that pretreatment of fenofibrate in PQ-stimulated cells increased Trx-2 expression, decreased the formation of p-Ask-1 and inhibited cytochrome c release. Cytochrome c release is an initiator of the main apoptotic pathway [66]. When cytochrome c is released from the mitochondria to cytosol, it binds to Apaf-1 and activates an apoptosis-related caspase cascade, consequently inducing PARP-1 cleavage leading to apoptosis [67]. We observed that fenofibrate treatment reduced the levels of cytosolic cytochrome c and the related caspase cascade in PQ-stimulated cells. In summary, our results indicated that fenofibrate could protect against oxidative stress-induced retinal/choroidal endothelial cell apoptosis by enhancing Trx-1 and Trx-2 expression, thereby suppressing Ask-1 activity, which in turn inhibits the activation of the subsequent apoptotic signaling pathways.

Our study has some limitations. It is an in vitro analysis, and the protective effects of fenofibrate and the underlying mechanisms need to be demonstrated with animal models. However, two large randomized controlled trials (FIELD and ACCORD study) have shown significant benefits of fenofibrate in patients with DR. Our results supported the assertion that fenofibrate can slow the progression of DR by modulating apoptosis- and stress-related signaling pathways.

## 5. Conclusions

Our study demonstrated that fenofibrate inhibited ROS accumulation, diminished mitochondrial dysfunction, as well as modulating several apoptotic and survival signal pathways in oxidative stress-induced RF/6A cells. The mechanism of action could be through enhancing Trxs expression and suppressing Ask-1 activity, which in turn inhibited the subsequent apoptotic signaling pathways. The anti-oxidative and anti-apoptotic beneficial effects of fenofibrate identified in this study may provide new insights into the design of therapeutic strategies concerning the imbalance between pro-apoptotic and survival pathways induced by oxidative stress in DR.
